# The Development of Mindful-Based Dance Movement Therapy Intervention for Chronic Pain: A Pilot Study With Chronic Headache Patients

**DOI:** 10.3389/fpsyg.2021.587923

**Published:** 2021-04-16

**Authors:** Indra Majore-Dusele, Vicky Karkou, Inga Millere

**Affiliations:** ^1^Department of Health Psychology and Pedagogy, Faculty of Public Health and Social Welfare, Riga Stradins University, Riga, Latvia; ^2^Research Centre for Arts and Wellbeing, Edge Hill University, Ormskirk, United Kingdom

**Keywords:** dance movement therapy, mindfulness, chronic pain, anxiety, depression

## Abstract

Chronic pain is of significant global concern. There is growing evidence that body–mind therapies and psychological approaches can contribute toward changing chronic pain perceptions. This is the first model described in the literature that combines a mindfulness-based approach with dance movement therapy and explores the potential psychological and pain-related changes for this client population. In this paper, the results from the pilot study are presented involving patients with chronic headache recruited in an outpatient rehabilitation setting.

**Methods:** In this pilot study, 29 patients (*n* = 29) with chronic headache were randomized to either the Mindful-Based Dance Movement Therapy (MBDMT) group or the waiting list control group (treatment as usual, TAU). The MBDMT group was offered 10 sessions in a clinical outpatient rehabilitation setting for 5 weeks. Data were collected pre- and post-intervention and 16 weeks after the intervention was finished. The Hospital Anxiety and Depression Scale (HADS), Patient Health Questionnaire−9 (PHQ-9), Five Facet Mindfulness Questionnaire (FFMQ), and Numeric Rating Scale (NRS) were used as outcome measures.

**Results:** The working model of MBDMT identifies nine therapeutic mechanisms (safe therapeutic environment, mindfulness skills, body awareness, relaxation/releasing, distancing and staying with discomfort, meaning making, self-regulation, acceptance and integration, creative process). Per-protocol analysis reveals statistically significant reduction of pain intensity and depression scores in favor of the MBDMT group, and these improvements were maintained in the follow-up assessment.

**Conclusions:** The results suggest that MBDMT is a feasible and promising therapy approach for chronic pain patients. The pilot study offered sufficient information and preliminary results in the desirable direction to enable the researchers to move to a randomized controlled trial (RCT) stage in order to establish the efficacy of the intervention.

**Clinical Trial Registration:** The study was registered in the www.researchregistry.com, registry (5483).

## Introduction

Chronic pain affects 20% of the worldwide population (Goldberg and McGee, 2011; Fayaz et al., 2016). In fact, 150 million Europeans suffer from moderate to severe chronic pain^1^. This debilitating condition affects the persons' physical, emotional, and social functioning, being a major source of suffering at the same time as it is a significant economic burden and challenge for health care systems^2^. Since 2019, a significant change has been made in the classification of chronic pain as the World Health Organization has adopted the new edition of International Classification of Diseases, 11th revision (ICD-11). ICD-11 will be the first classification to include chronic pain as a health condition in its own right. This new classification differentiates chronic primary pain from chronic secondary pain, with the former referring to pain in one or more anatomical regions that persists or recurs for longer than 3 months. Furthermore, it recognizes that chronic primary pain is closely linked with significant emotional distress (anxiety, anger/frustration, or depressed mood) and it interferes with the activities of daily life and participation in social roles in ways in which cannot be accounted for by another chronic pain condition^3^. The new classification system is expected to promote research on the etiology and pathophysiology of these conditions and to improve access to multimodal care for all patients with chronic pain (Treede et al., 2019). Several emotional distress factors are regarded as mediators in the “chronification” of pain, that is, persistent pain with characteristic pain behavior and resistance to therapeutic intervention (Borsook et al., 2018). These factors include pain catastrophizing, anxiety, and fear of pain and helplessness (Keefe et al., 2004). Depression is also seen as a serious risk factor in the development of debilitating pain (Hülsebusch et al., 2016). The psychological characteristics of chronic pain patients can involve abuse and neglect experiences in childhood (Davis et al., 2005), counter-dependency traits, and alexithymia (Ak et al., 2004), with depression working as a mediator between chronic pain and alexithymia (Saariaho et al., 2013). These psycho-emotional characteristics of chronic pain patients make this patient population quite a heterogeneous group. The large-scale cross-sectional study of chronic pain patients identified four subgroups. They differ in aspects of pain intensity, duration and spreading, psychological strain, and social distress including lack of social support. Research confirms that all three components–bio-psycho-social–are important in chronic pain, but different constellations of these components form subgroups with different needs in pain treatment and indicate the need to design “tailor-made” interventions (Bäckryd et al., 2018).

Epidemiology studies show that the prevalence of pain is higher in women: chronic primary headache, in particular, affects more women than men (Jiménez-Sánchez et al., 2012; Allais et al., 2020). Middle age (40–59 years old) has been reported as high risk: people in this group appear to be less satisfied with their social life and are more often diagnosed with fibromyalgia. The older adult group reported higher life quality scores, higher levels of satisfaction with marital and social life, and better mood, but they face more comorbidities and longer periods of pain (Rustøen et al., 2005). Seniors also report lower levels of pain severity and pain interference and greater levels of perceived control over pain in comparison with younger people suffering from headaches (Lachapelle and Hadjistavropoulos, 2005).

Treatment strategies, which are regarded as clinically effective and cost efficient, tend to be multidisciplinary and are based on the biopsychosocial model of pain (Gatchel et al., 2007), acknowledging psychological risk factors (Nicholas et al., 2011) and offering holistic approaches to multimodal pain management (Kress et al., 2015). The biopsychosocial model of pain recognizes that pain has three facets: cognitive-evaluative, sensory-discriminative, and affective-motivational aspects (Melzack, 1999). All of these need to be included in a treatment package. From a wide range of psychological therapies, Cognitive Behavioral Therapy (CBT) and Acceptance and Commitment Therapy (ACT) present the strongest evidence for decreasing depression, pain-related anxiety, and catastrophizing and for increasing self-efficacy (Williams et al., 2012; Veehof et al., 2016). In addition, studies on Mindfulness-Based Stress Reduction (MBSR) program report decreasing pain intensity and disability (Cramer et al., 2012). MBSR was a program created and introduced by John Kabat-Zinn, who defines mindfulness as “the awareness that emerges through paying attention on purpose, in the present moment, and non-judgmentally to the unfolding of experience moment to moment” (Kabat-Zinn, 2003, p. 145). Mindfulness allows patients to relate to their physical and psychological symptoms in a different, more skillful way, with a positive effect on developing a realistic sense of control and appropriate strategies in becoming adaptive (Kabat-Zinn et al., 1986; Grossman et al., 2010). Other Mindfulness-Based Interventions (MBIs) show a positive impact on perceived pain control, pain acceptance, and quality of life (Bawa et al., 2015). In MBIs, the indirect effect on pain is due to an increased acceptance; this buffers the intensity of perceived pain as a stressful event (Shapiro et al., 2006). Analysis of the content of MBIs reveals meditation practice, exercises that support the change of habits, simple yoga exercises, and psychoeducation as some of the main structural components of the intervention (Majore-Dušele et al., 2018). Furthermore, monitoring attention and acceptance are the central mechanisms in mindfulness training programs; these interact to improve stress, affect, and health outcomes (Lindsay and Creswell, 2017).

However, these interventions are limited in their capacity to approach the person as a whole, prioritizing often the cognitive domain as the route through which change may occur. They require considerable cognitive and linguistic skills from the client/patient and acknowledgment of psychological difficulties and mental health concerns in their physical symptoms. As chronic pain patients experience the physical nature of their symptoms and may perceive their mental health concerns as stigma, they may refuse cognitive-based interventions (Payne and Brooks, 2018). With chronic pain being situated in the body, there is a danger that cognitive-based interventions are missing the opportunity to validate the physical suffering and work with the body in order to find therapeutic solutions. They also make limited use of the connection between the body and the mind as a means through which change may occur, ignoring the role movement can play as a holistic, creative, and thus therapeutic tool. For a body-based condition, such as chronic pain, there may be value in the development of a body–mind intervention with a holistic/creative character. Dance Movement Therapy (DMT) is one such intervention.

DMT is defined as “the therapeutic use of movement to further the emotional, cognitive, physical, spiritual, and social integration of the individual.” Somatic awareness and kinesthetic empathy, movement as creative self-expression and dance as non-verbal interaction, are the core components of DMT (European Association of Dance Movement Therapy 2013, p.1)^4^.

There is growing evidence that DMT, a creative body–mind form of psychotherapy, may have a positive psychological impact on the psychological states of patients with somatic concerns. Dance and movement, being key elements in the therapeutic alliance between patient and therapist, can provide the means for the self-expression and communication of unspoken concerns among this client population. The latest meta-analyses show that DMT is helpful in health-related psychological outcomes, improving well-being, mood, affect, quality of life, body image, and interpersonal competence and reducing clinical symptoms, such as anxiety and depression, for different patient groups (Koch et al., 2014, 2019; Meekums et al., 2015; Karkou et al., 2019). DMT has shown promising improvements in functioning for fibromyalgia patients (Bojner-Horwitz et al., 2003) and patients with medically unexplained symptoms (Payne and Brooks, 2016, 2017, 2020). However, until now, there has been limited research on the effectiveness of DMT with patients suffering chronic pain. One of the few studies with this client population comes from Shim (2015b) and Shim et al. (2017) who found that the 10-week DMT process increased resilience, decreased “kinesiophobia” (i.e., fear of movement), and showed a beneficial impact on pain intensity.

Regardless of the type of psychological therapy, there are arguments that the mechanisms of change in each therapeutic intervention need to be considered specifically for each client population (Kazdin, 2009; Burns, 2016). In pain treatment, *mindfulness, acceptance*, and *self-efficacy* are recognized mechanisms in pain regulation (Turner et al., 2016). In DMT work with chronic pain patients, increasing *self-compassion* has been set as a main principle of the work, which is facilitated through alternating between states of acceptance and inspiration (Erber, 2015). The BodyMind Approach® (TBMA) (an approach that has been derived from DMT) has identified five key factors as responsible for successful self-management of patients with medically unexplained symptoms. These are: body with mind connections, importance of the facilitator, positive benefits, preparedness for change, self-acceptance, and compassion (Payne and Brooks, 2020). The DMT model for building resilience in pain patients by Shim et al. (2017) recognizes the mechanisms of *activating self-efficacy, connecting to self*, *connecting to others, enhancing emotional intelligence*, and *reframing* as important. In earlier models of DMT for chronic pain patients (Shim, 2015a), four therapeutic factors were identified: *kinesthetic awareness* (articulation, noticing, widening), *enactment* (mobilization and motivation, kinesthetic imagining, reinforcement and reframing), *expressivity* (externalizing and symbolization, emotional restoration and management, creativity and ability to play), and *making connections* (mind–body integration, meaning-making and identity reconstruction, interpersonal connection).

Despite the discussion in DMT literature that mindfulness is an important component of DMT (Koch et al., 2019), there has been no prior study that integrates these two approaches and explores the potential psychological and pain-related changes for this client population. In this paper, the development of the working model of the Mindful-Based Dance Movement Therapy (MBDMT) is presented, along with the results from the pilot study that has been completed, before conducting a large randomized controlled trial (RCT) that will examine the efficacy of MBDMT for chronic pain patient population.

The objectives of the pilot study were to: (1) establish recruitment and follow-up processes, (2) explore the initial outcome results of MBDMT intervention, (3) examine intervention acceptability and participant adherence, and (4) test adherence to the MBDMT protocol. This current paper will present and discuss how these objectives were met.

## Methods

### Recruitment Procedure and Participants

The present study recruited patients with chronic headache (tension-type headache and/or migraine with or without aura, diagnosed by a neurologist). Patients with chronic headache were prioritized in this study because of easy access to this population in the first instance. Inclusion and exclusion criteria were established prior to the commencement of the study. Inclusion criteria for the study were: (1) primary headache lasting more than 3 months (headaches needed to be the primary cause for seeking medical help), (2) being between 20 and 55 years old, and (3) increased depression and/or anxiety measures [Patient Health Questionnaire−9 (PHQ-9) ≥5; Hospital Anxiety and Depression Scale (HADS) ≥7]. Exclusion criteria were: (1) patients who had a disease based on an infectious process, autoimmune or metabolic pathology, traumatic injury, neoplastic process (primary tumor or metastasis), or internal organ pathology that can be connected with pain; (2) movement limitations not related to the diagnosis of chronic pain (e.g., cerebral palsy, spinal injuries); and (3) pregnancy.

The recruitment strategy involved: (i) the neurologists of the rehabilitation setting who were informed at their quarterly meeting about the aims and structure of the intervention and the inclusion and exclusion criteria of the pilot study and (ii) participation in the research and DMT groups was advertised through social media in a closed group of the headache patient's association. Interested participants allowed their neurologist to send contact information to the principal investigator (first author of this paper). Alternatively, they were given the option to contact the principal investigator themselves through email or SMS. The principal investigator made an initial phone call to each interested individual to inform them about the study, to answer any questions, and to gain verbal consent for participation in the study. Potential participants were informed about randomization and were also informed that members of the control group could participate in the DMT group after the intervention group process was complete. Following this, all participants were sent by email written information about the study, the consent form, and all measures for the baseline assessment. The recruitment process and all aspects of the study gained ethical approval by the Ethics Committee of Riga Stradins University (02/28/2019, no. 6-3/2/43).

Over the 10 weeks of the recruitment period (August–November 2019), 39 women (*n* = 39) expressed interest in participating in the study; 19 were recommended for participation by their neurologists, whereas the other 20 were recruited through the headache patient's association. Although no prevalence or restrictions were identified in the inclusion criteria regarding sex, only female participants came forward to participate in the study. After the baseline assessment, 29 patients (*n* = 29) were eligible for inclusion. These patients were randomly assigned to the MBDMT intervention or the waiting list control group using the random number generator that produced two sets of 15 unique numbers from 1 to 30.

All participants in the study received treatment as usual (TAU), continuing in their rehabilitation outpatient setting. TAU for chronic headache patients was pharmacological treatment ordered by a neurologist along with physical and/or physiotherapy input (Table 1).

**Table 1:** Baseline participant demographic and clinical characteristics in the intervention and control groups.

**Characteristics**	**Intervention group**	**Control group**	***p***
	**(*n =* 15)**	**(*n =* 14)**	
Age, M (SD)	40.9 (6.97)	32.2 (4.98)	0.001^a^
**Localization of pain**	***N*** **(%)**	***N*** **(%)**	
Headache, migraine	15 (100)	14 (100)	
Lower back pain	8 (53.3)	5 (35.7)	0.340^b^
Musculoskeletal	7 (46.7)	2 (14.3)	0.109^c^
Fibromyalgia	2 (13.3)	1 (6.7)	1.0^c^
**Duration of pain**			0.431^c^
Until 1 year	1 (6.7)	2 (14.3)	
1–3 years	2 (13.3)	0 (0)	
3–5 years	0 (0)	1 (7.1)	
5–10 years	2 (13.3)	4 (28.6)	
More than 10 years	10 (66.7)	7 (50.00)	
**Pain control strategies**			
Medication	14 (93.3)	13 (85.7)	0.598^c^
Physiotherapy/massage	9 (60.0)	5 (35.7)	0.191^b^
Daily exercise	3 (20.0)	2 (14.3)	1.0^c^
Physical procedures	5 (33.3)	4 (28.6)	1.0^c^
Relaxation/meditation	2 (13.3)	1 (7.1)	1.0^c^

a Mann–Whitney U-test;

b Pearson Chi-Square;

c *Fisher Exact test*.

### Outcome Measures

Assessments were performed at baseline (T1), post-treatment (T2; 2 months after baseline), and 4 months post-intervention (T3; 4-month follow-up).

*Demographic information* was collected through a self-completed questionnaire created by the researcher, which gathered information about gender, age, and pain characteristics—pain duration, pain etiology, and types of pain control strategies.

*A Numeric Rating Scale (NRS)* was used as a scale for pain intensity measurement. This is an 11-point scale where the end points are the extremes of no pain (0) and worst possible pain (10). The NRS can be graphically or verbally presented and can be self-assessed. This method of assessing pain is widely recommended as a core outcome measure in clinical trials of chronic pain treatment (Farrar et al., 2001; Dworkin et al., 2005).

*The HADS* (Zigmond and Snaith, 1983) evaluates the severity of anxiety and depression symptoms in non-psychiatric inpatients. It is composed of seven items that assess anxiety symptoms and seven for depression symptoms. Each item contains a scale of 4 points (from 0 to 3) with total scores ranging from 0 to 21 for anxiety and depression in three categorical levels: normal (0–7), borderline abnormal (8–10), and abnormal (11–21). Higher scores mean greater severity. Psychometric properties for the Latvian version showed a good internal consistency (Cronbach's alpha 0.892) (Šmite and Ancāne, 2010). In the present study, the internal consistency at T1 was Cronbach's α = 0.82 for depression scale and α = 0.80 for anxiety scale, at T2 α = 0.79 and α = 0.82, and at T3 α = 0.74 and α = 0.73.

*The PHQ-9* (Kroenke and Spitzer, 2002) is a self-administered dual-purpose instrument that can establish provisional depressive disorder diagnoses as well as grade depressive symptom severity. Each of the nine items is scored from 0 to 3, providing a severity score ranging from 0 to 27. Severity of depression was assessed by the PHQ-9 depression severity score and graded as none/minimal (0–4), mild (5–9), moderate (10–14), moderately severe (15–19), and severe (20–27). In the present study, the internal consistency of T1 data was Cronbach's α = 0.79, T2 α = 0.74, and T3 α = 0.70.

*The Five Facet Mindfulness Questionnaire* (FFMQ; Baer et al., 2006) is composed of 39 items. Each item is rated on a 5-point Likert scale (1 = “never or very rarely true”; 5 = “very often or always true”). Five scales: “Observing” is the ability to notice or attend to internal and external experiences, such as sensations, thoughts, or emotions. “Describing” means to label internal experiences with words. “Acting with awareness” refers to focusing on one's activities in the moment as opposed to behaving mechanically. “Non-judging of inner experience” means taking a non-evaluative stance toward thoughts and feelings, and “non-reactivity to inner experience” refers to allowing thoughts and feelings to come and go, without getting caught up in or carried away by them. Higher scores on the FFMQ reflect greater mindfulness skills. The Latvian version of this scale has shown good reliability and internal consistency (Cronbach's alpha 0.9; Majors, 2013). In the present study, the internal consistency of T1 data was Cronbach's α = 0.89, T2 α = 0.86, and T3 α = 0.91.

### Intervention

**Figure 1:**
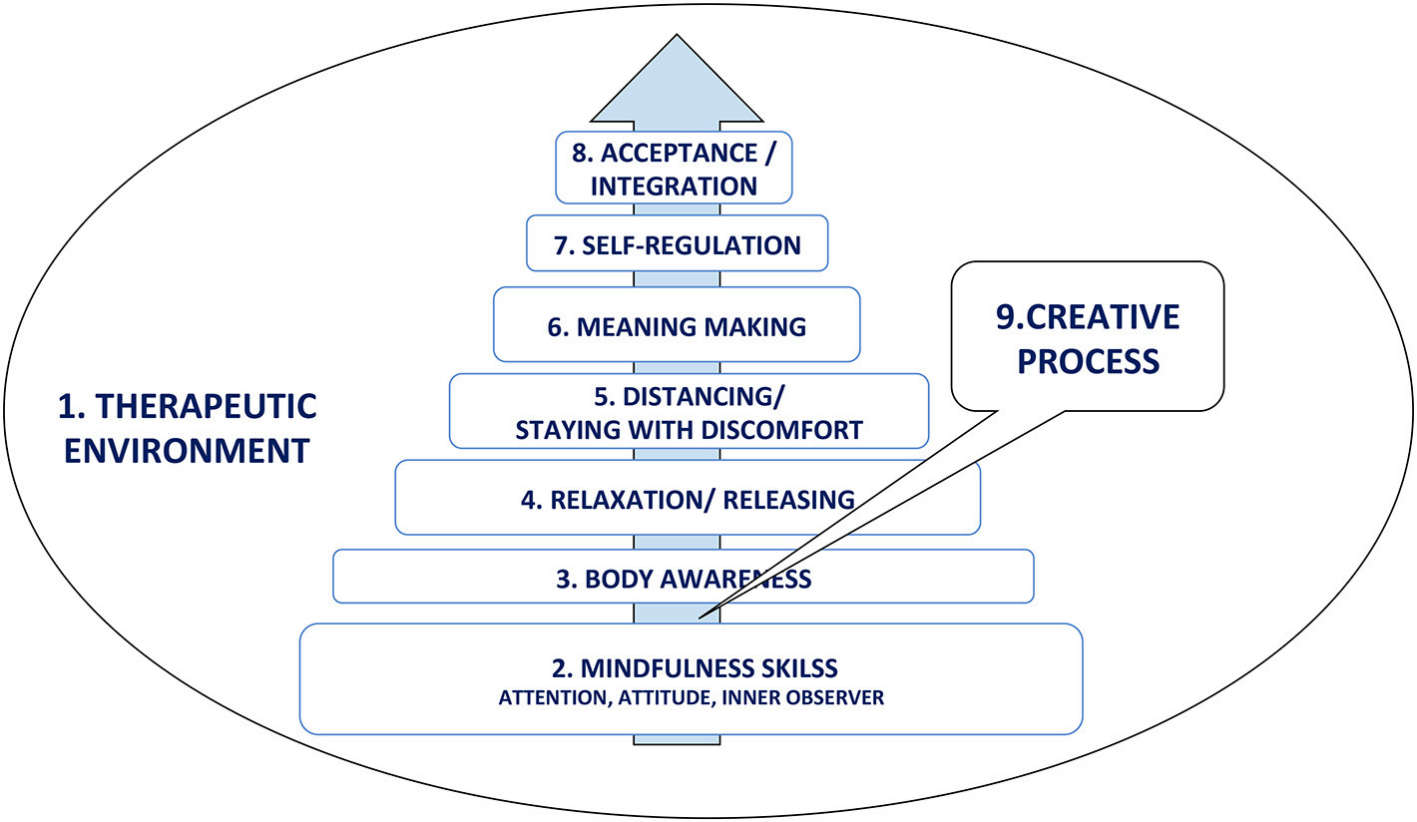
Mindful-based dance movement therapy intervention model.

The MBDMT intervention model used in this study was grounded on the contribution of five expert informants (Figure 1). It involved nine therapeutic mechanisms/components of change, which were structured in a developmental process with each component supporting the next one.

1. *Safe therapeutic environment*. This is a common factor in any counseling or psychotherapy process. Its main characteristics are warm relationships built on empathy, support, validation, and the acceptance of the patient as he/she is—emotionally, physically, and cognitively. These are also known as the person-centered facilitative conditions (Lambert and Barley, 2001). The therapeutic environment is a safe holding space for the other factors in the therapy process.
2. *Mindfulness skills*. The patient will be supported to focus and regulate their attention to different realms of perception: their own sensations, emotions, thoughts, and images, and to cultivate kindness, compassion, and a non-judgmental attitude toward their self. The development of mindfulness skills will serve as gateway into the creative process.
3. *Body awareness*. The patient will pay attention toward their body sensations, not reacting negatively or emotionally but with an explorative interest. Attention toward interaction between sensation, emotions, thoughts, and experience of body–mind connectivity.
4. *Relaxation/releasing*. Through creative activities, verbalization, and sharing, the patient will express themselves safely and release physical tension. This will open them up for new experiences and perceptions of themselves and others.
5. *Distancing and staying with discomfort*. Instead of persisting with fear and avoidance reactions, the patient will use creative tools (props) and aesthetic distance to focus on pain. This will help them to explore their relationship with their symptoms and to build tolerance and the ability to manage their discomfort.
6. *Meaning making*. The patient will be invited to respond to the question: “why is this happening to me?” In-depth exploration of symptoms in the context of one's personal life and possible insights into the cause–effect relationship of symptom development.
7. *Self-regulation*. The patient will practice self-management, recognizing and releasing physical tension and dealing with any emotionally intense feelings that may arise.
8. *Acceptance and integration*. As a result of the therapeutic process, the patient may gain a deeper understanding of themselves, as well as an acceptance of their physical and emotional states.
9. The *Creative process* permeates all of the above factors in the model, as they are delivered through creative activities. Imagery, symbolism, and movement metaphors are core aspects of DMT.

The therapy model described above shows that MBDMT is a purposefully organized therapeutic process where, through the use of creative activities, the development of mindfulness skills, and an exploration of bidirectional processes, the relationship between body and mind is explored, and self-management and self-regulation skills are learned (Majore-Dušele et al., 2019).

The protocol of the MBDMT intervention is organized as a short-term focused therapeutic process taking into the account the short-term organization structure of rehabilitation settings in Latvia. It includes 10 sessions, twice a week, for a 5-week period. Each session lasts 90 min and follows a similar structure: (1) check-in and physical warm-up; (2) body-scan (sitting, lying, standing, or walking); (3) work with themes—safety, pleasure, personal borders, body–mind connection, relationship with pain, and resources; and (4) closure and homework.

Two therapeutic groups took place, with seven/eight participants in each group. Three participants were lost early in the process, leaving a group of 12 (*n* = 12) from whom the data were derived. The groups were facilitated by a dance movement therapist in training (3rd year professional Masters student in arts therapies with specialization in dance movement). The facilitator had been licensed as a psychotherapist prior to training as a dance movement therapist, had more than 5 years practice working with mental health issues and adult groups, had previous mindfulness practice training (8 weeks program), received training in delivering the MBDMT intervention (24 h), and had supervision during the intervention period by a qualified supervisor. MBDMT intervention training included personal experience with the MBDMT model and facilitating skills training.

### Analysis

Data were analyzed using the Statistical Package for Social Science, version 23 (SPSS-23, IBM SPSS Statistics for Macintosh, version 23.0; IBM Corp., Armonk, NY, USA). Descriptive statistics were used to present the characteristics of the sample. The Kolmogorov–Smirnov test indicated that the distribution departed from normality and the small sample size limited the power of statistical analysis, so the Mann–Whitney *U*-test was used to analyze baseline group differences of the interval data (i.e., socio-demographic and clinical characteristics), whereas the Pearson Chi-Square test or Fisher's Exact Test was used for categorical variables (localization and duration of pain and pain control strategies). The Mann–Whitney *U*-test was also used to conduct a between-group comparison of the change in scores from baseline (T1) to the post-treatment (T2) and follow-up (T3) for all the outcome measures with *p* < 0.05 being the accepted level of statistical significance. Although medians (interquartile range) were used for the calculation of averages, means, and standard deviations were also presented. Taking into account the small sample size of this pilot study, the statistical analysis was performed Per Protocol (PP) instead of performing Intention To Treat (ITT) analysis. PP included patients who completed the intervention according to the protocol only along with data collected from the control group. In all cases, missing data were not included in the calculation.

The Reliable Change Index (RCI; Jacobson and Truax, 1991) was calculated for PHQ-9, HADS, and pain measures to assess the impact of the intervention at the individual level. The RCI was used as an indicator of clinical significance of change because it allowed a determination of whether an individual change score (between pre-intervention and post-intervention assessment) was significantly greater than a difference that could have occurred due to random measurement error alone (Guhn et al., 2014). Cut-off points dividing the clinical and non-clinical patient groups were ≥10 for PHQ-9 and ≥8 for HADS as the most frequently recommended ones in the literature (Bjelland et al., 2002; Hansson et al., 2009; Manea et al., 2012). Participants whose PHQ-9 and HADS scores were below the cut-off point at the pre-treatment measurement (T1) were excluded from the RCI calculation. The RCI was calculated at 95% confidence. If a participant's RCI was below −1.96 and passed the cut-off point, the participant was classified as *recovered*. If the RCI was below −1.96 but did not pass the cut-off point, the participant was classified as *improved*. If the participant's RCI was between −1.96 and 1.96, the participant was classified as *unchanged*. If the participant's RCI was above 1.96, the participant was classified as *deteriorated*. For pain measures, the RCI was calculated based on the minimal clinically important difference being 2 points on a 10-point rating scale as reported in a previous study (Hägg et al., 2003).

Power calculation was performed for the primary outcome measure—depression (PHQ-9), to detect the adequate sample size for future RCT.

## Results

### Recruitment and Follow-Up Feasibility

**Figure 2:**
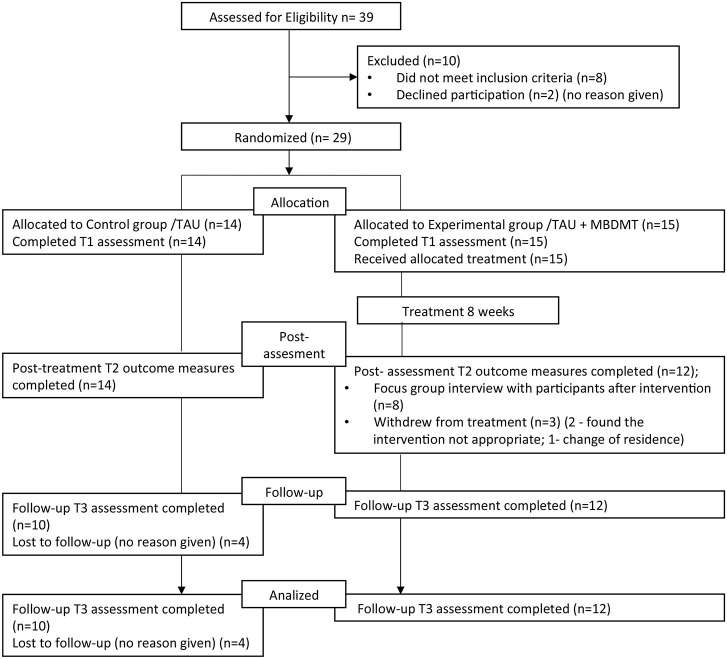
Recruitment flow diagramm.

During the 2.5-month enrollment period, 39 women expressed an interest in participating in the study, and 29 met the eligibility criteria. Both recruitment strategies (i) by the neurologists and (ii) by headache patients' association were similarly effective, attracting 50 and 50% of the study participants. As the study flow diagram shows (see Figure 2), post-treatment measures used immediately after the intervention were completed by all the members of the control group and all those from the intervention group who attended the whole duration of the program. Three participants who did not complete therapy discontinued early in the study. Follow-up measures were completed by 100% of the intervention group completers and 71% (10/14) of the control group.

### Sample Characteristics

All the participants in the study were female and between 26 and 55 years old (M = 36.7, SD = 7.4). Of the patients, 56.7% had suffered from pain (headache, migraine) for more than 10 years. Furthermore, 60% of the patients suffered from other types of pain in addition to headache and migraine. The participants in the intervention and control groups were compared by demographic characteristics in order to establish whether there were differences between them at baseline. The participants in the intervention group were older (M = 40.9, SD = 6.9) than the participants in the control group (M = 32.6, SD = 5.0). There were no differences between the two groups in the duration of pain experience and variabilities of pain type or pain control strategies (Table 1).

### Treatment Outcome Results: Changes in Intervention Group vs. Control Group

The means, standard deviations, and medians of the outcome measures, as well as between group comparison of the baseline/post (T1/T2) and baseline/follow-up (T1/T3) change scores, are presented in Table 2 at three measurement points. The results indicate that, at baseline (T1), there were no statistically significant differences between the groups in the measurements of pain, PHQ-9, HADS, or FFMQ. For each of the scales with clinical cut-offs, the selected population baseline mean was above the clinical cut-offs, PHQ-9 (M = 8.1, SD = 4.3), HADS-A (M = 9.8, SD = 4.1), and HADS-D (M = 5.34, 3.8), respectively, indicating mild to moderate symptoms of anxiety and depression for both groups.

**Table 2:** Means, standard deviations, and medians of clinical outcome measures between group comparison of the pre/post and pre/follow-up change scores.

**Variables**		**Intervention group (*****n =*** **12)**		**Control group (*****n =*** **14)**		**Mann–Whitney** ***U*****/*****p*****-value**		
**(Cronbach's α)**	**Time**	**Mean (SD)**	**Mdn (IQR)**	**Mean (SD)**	**Mdn (IQR)**			
Pain (NRS)	T1	6.00 (1.28)	6 (2)	6.33 (1.22)	6 (3)	102.5/0.35		
	T2	4.83 (1.34)	5 (1)	6.22 (1.56)	6 (2)			
	T3	4.67 (1.44)	4 (2)	6.00 (1.23)	6 (2)			
	(Δ) T1 to T2	−1.17 (0.83)	−1.00 (1.75)	−0.11 (1.17)	0.00 (1.5)		39.5/0.02^*^	
	(Δ) T1 to T3	−1.33 (0.98)	−1.5 (1.75)	−0.33 (1.0)	0.00 (1.5)			26.5/0.04^*^
PHQ-9	T1	8.08 (4.64)	6 (5)	8.67 (4.53)	8 (6)	86.00/0.92		
(0.80)	T2	4.58 (2.61)	4.5 (4)	8.33 (4.21)	9 (6)			
	T3	4.17 (1.75)	4.5 (2)	6.78 (3.03)	8 (6)			
	(Δ) T1 to T2	−3.5 (6.19)	−2.00 (3.00)	−0.33 (2.96)	−1 (2)		40.5/0.02^*^	
	(Δ) T1 to T3	−3.91 (4.12)	−2.5 (3.75)	−1.89 (3.59)	−2 (3.5)			35.5/0.18
HADS-Anx	T1	10.83 (4.39)	11.5 (6)	9.22 (3.89)	9 (5)	56.00/0.15		
(0.82)	T2	8.08 (3.91)	7.5 (7)	8.56 (3.21)	9 (5)			
	T3	8.17 (2.72)	8.5 (3)	9.22 (3.93)	9 (6)			
	(Δ) T1 to T2	−2.75 (3.67)	−2 (3.75)	−0.67 (2.24)	0.00 (3)		121.0/0.06	
	(Δ) T1 to T3	−2.67 (3.31)	−4 (4.75)	0.00 (3.46)	−1 (7)			78.5/0.08
HADS-Depr	T1	6.25 (3.72)	5 (5)	4.57 (3.81)	4.5 (5.8)	64.00/0.30		
(0.80)	T2	4.75 (3.17)	5 (4)	4.22 (2.77)	4 (5)			
	T3	4.42 (2.61)	4 (3)	3.44 (2.83)	3 (6)			
	(Δ) T1 to T2	−1.5 (3.70)	−1.5 (3.75)	0.44 (1.94)	0.00 (6)		118.5/0.07	
	(Δ) T1 to T3	−1.83 (2.72)	−1 (4)	−0.33 (2.00)	0.00 (3)			76.5/0.11
FFMQ_full scale	T1	129.25 (20.04)	127 (32.3)	138.67 (15.35)	141 (49)	92.5/0.67		
(0.89)	T2	136.92 (17.05)	135 (15)	137.11 (9.21)	136 (10.5)			
	T3	134.75 (18.69)	136.5 (22.3)	137.11 (21.99)	141 (31.5)			
	(Δ) T1 to T2	−7.67 (22.69)	−2.5 (21.25)	1.55 (7.52)	2 (8.5)		108.5/0.21	
	(Δ) T1 to T3	−5.5 (16.63)	−4 (26)	1.55 (10.97)	2 (16.5)			65.00/0.43

*IG—intervention group; CG—control group; (Δ) was computed by subtracting pretest from posttest and pretest from follow-up scores; positive values signify an increase, and negative values signify a decrease in the dependent measure. Results from NRS, Numeric Rating Scale; PHQ-9, Patient Health Questionnaire−9; HADS, Hospital Anxiety and Depression Scale; FFMQ, Five Facet Mindfulness Questionnaire—full scales results*.

* *p < 0.05*.

A Mann–Whitney *U*-test was conducted to look for statistically significant differences in the reduction of pain, depression, and anxiety scores across treatment conditions. The test identifies a significant difference in the change score (T1/T2) of perceived pain between the MBDMT group (Mdn = −1) and the control group (Mdn = 0.00), *U* = 128.5, *p* = 0.02. The change remained statistically significant in T1/T3 between the intervention group (Mdn = −1.5) and the control group (Mdn = 0.00), *U* = 81.5, *p* = 0.04.

The reduction of PHQ-9 scores indicates that there were changes in symptoms for the intervention group (Mdn = −2), which was different from the control group in a statistically significant way (Mdn = −1), *U* = 40.5, *p* = 0.02 in T1/T2 comparison. The difference was not statistically significant when T1/T3 was calculated, *U* = 35.5, *p* = 0.18.

On HADS measures, the change score for anxiety and depression was greater for the control group in both post-intervention time points with reduced scores post-intervention than for the MBDMT intervention group, but this change did not reach statistical significance (HADS-A, T1/T2: Mdn = −2 vs. Mdn = 0.00, *U* = 121.00, *p* = 0.06; T1/T3: Mdn = −4 vs. Mdn = −1, *U* = 78.5, *p* = 0.08; HADS-D, T1/T2: Mdn = −1.5 vs. Mdn = 0.00, *U* = 118.5, *p* = 0.07; T1/T3: Mdn = −1 vs. Mdn = 0.00, *U* = 76.5, *p* = 0.11).

No significant differences between the groups were found for FFMQ scores as indicated in Table 2.

With reference to Table 3, 50% (6/12) of the intervention group participants experienced a reliable change in pain reduction of at least 2 points. For the other 50%, their pain level stayed unchanged. For 92% (11/12) of the participants, their anxiety level reached the clinical cut-off point at baseline for HADS-A, but follow-up measures demonstrated reliable improvement for 73% (8/11) of the participants. Furthermore, 28% (3/11) of the participants' anxiety levels changed from clinical to non-clinical population valuables. Only four participants' depression levels reached clinical cut-off points at baseline by both measures PHQ-9 and HADS-D, but at follow-up, three of these patients (75%) demonstrated a reliable improvement with regard to depression, changing from the clinical to the non-clinical population. One patient, from the intervention group included in RCI calculations, demonstrated a significant increase in anxiety.

**Table 3:** Reliable change of the intervention group using 95% CI for outcome measures pre-intervention to follow-up.

**Outcome variables**	**Recovered**	**Improved**	**Deteriorated**	**Unchanged**
Pain (NRS) *n =* 12	0%	50% (6/12)	0%	50% (6/12)
PHQ-9 *n =* 5	80% (4/5)	0%	0%	20% (1/5)
HADS-D *n =* 4	75% (3/4)	25% (1/4)	0%	0%
HADS-A *n =* 11	28% (3/11)	45% (5/11)	9% (1/11)	18% (2/11)

As this pilot study demonstrated statistically significant decrease in depression, the PHQ-9 measure of depression was used as a primary outcome of the intervention. Based upon the follow-up data, using the means and standard deviations, sample size calculation for a larger RCT was performed. Calculation for continuous outcome superiority trial with 80% power at an alpha level of 0.05 suggested that a total sample of 86 participants were needed to detect clinically important difference between means. To allow for attrition between end of trial and follow-up, the aim was to recruit 120 patients for an RCT.

### Intervention Acceptability, Participant Adherence, and Satisfaction

Of the 15 patients who began to participate in the MBDMT group, 12 completed the therapy course and attended at least eight sessions. The three participants who did not complete the treatment discontinued early in the study: one moved to a new residence, and two concluded after the first session that the intervention was not appropriate for them. Four patients completed all 10 sessions, 3 patients completed 9 sessions, and 5 patients completed 8 sessions.

### Adherence Relative to MBDMT Protocol

Adherence to the treatment protocol from the dance movement therapist in training was evaluated through a selection of video recordings from the sessions, looking at different stages of the work (warm-up, working, and closing stages). This was also discussed during regular supervision sessions. Substantial diversion from the protocol was not observed. The nine working mechanisms that informed the structure of the intervention were found to be logical and supportive of the development of the group therapy process. There was, however, some difficulty in the timing of the sessions: the therapist in training found that more time was needed at the beginning and end of the sessions to allow for the participants' verbal discussion.

On the whole, the treatment protocol was easy to follow and responsive to group dynamics offering alternative choices of techniques.

## Discussion

This pilot study suggested that MBDMT was a useful intervention for reducing the pain, depression, and anxiety symptoms of chronic pain patients participating in the study. The results indicate that offering a DMT group intervention in addition to the usual medical treatment of chronic pain improved the psychological aspects and reduced pain more than just medical treatment and physiotherapy. Viewing the results of the present study within the context of other DMT and mindfulness-based intervention studies, the study offers an interesting addition to the literature, especially with regard to pain reduction. The results from systematic reviews show that MBIs are inconsistent in terms of their effect on pain reduction. The results vary from medium to weak effectiveness, but do reveal a positive impact on perceived pain control with a moderate effect size (g = 0.58) (Cramer et al., 2012; Bawa et al., 2015). Note that the goal of MBIs is not to reduce the intensity of the pain but to improve the patient's functioning and reduce their general distress; being mindful has a therapeutic value in its own right (Reiner et al., 2013).

The results from this MBDMT study, however, do suggest a statistically significant reduction in pain intensity, in contrast with the mindfulness measure where no significant change was observed. While pain reduction is not the primary goal of the MBDMT intervention, the pain experience of the patient is validated, and the relationship with pain is addressed in the work phase of the therapeutic process. For 50% of the MBDMT participants, pain reduction was at least 2 points on the NRS scale; this is considered to be a reliable and clinically important change (Farrar et al., 2001). These results are consistent with an earlier research in DMT for chronic pain patients, in which the theme of working with the meaning of pain was part of the therapeutic process, and the reduction in pain intensity was observed (Shim et al., 2017).

The MBDMT intervention preliminary results also suggest that reduction in depression and anxiety symptoms is possible at least for the small sample of participants with chronic pain involved in this study. Individual analysis of reliable change for the intervention group indicated reliable improvement in anxiety symptoms for 73% of the patients. For those four patients with moderate and severe depression, improvement was clinically significant, allowing them to move to a non-clinical population. These results are consistent with previous research studies with a similar patient group with medically unexplained symptoms, including medically unexplained pain, where 65% of the participants involved in a (TBMA) group demonstrated reliable improvement on depression or anxiety measures (Payne and Brooks, 2017; Payne et al., 2017). These results can also support the results from a large-scale clinical trial on DMT and depression (Hyvönen et al., 2020) and meta-analyses on DMT for depression (Meekums et al., 2015; Karkou et al., 2019). In addition, potentially, they add to the results from generic meta-analyses on DMT (Koch et al., 2019). The latter study argues that DMT can improve psychological conditions by decreasing depression and anxiety levels for patients with somatic concerns. The results from the current study suggest that this was also true for the participants of the MBDMT group.

Findings from this current study may be biased, due to the small sample size and the possibility of a type II error (low power to detect true effects). However, it is important to mention that the follow-up measurement (T3) took place 2 weeks after the beginning of the 2019 coronavirus disease (COVID-19) pandemic in Latvia (March 2020), and that an overall atmosphere of fear and uncertainty may have influenced the participants' psycho-emotional state. This, in turn, may have been reflected in the results of the follow-up anxiety, depression, and also pain measures. In addition, it is important to acknowledge that 60% of the study participants had comorbid pain states, which may have influenced the study results. The comorbidity of other pain and mental states (depression and anxiety) is characteristic of women with migraine diagnosis and has been reported in previous chronic pain studies (Allais et al., 2020; Xu et al., 2020), suggesting that the mechanism of central sensitization may be a substrate or consequence of comorbidity (Ashina et al., 2018).

Patients found the experience of being in the MBDMT group to be useful. This was reflected in the acceptability of the intervention by the participants, which was evaluated as good; 80% of the participants assigned to the treatment group stayed in the process and attended at least 8 out of 10 therapy sessions. The literature suggests that other body-based interventions also show high completion rates. TBMA, for example, reported a completion rate of 95% (Payne and Brooks, 2017), and the resilience-building DMT approach for chronic pain patients was evaluated as helpful and supporting of a body–mind orientated approach by 68% of the participants (Shim et al., 2017).

Still, in the current study, since two patients did not find the intervention appropriate for them and dropped out of the intervention group after the first therapy session, an assessment of the intervention's suitability is needed. Although statistical tests showed no significant difference in baseline measures between those dropping out and those completing the intervention, HADS anxiety scores indicate that there were some differences worthy of further analysis. The mean score of anxiety (9.95) for the group completing the intervention can be seen as borderline with the mean score of the dropout group (14.00) being a clear outlier. It is possible that patients with the high anxiety scores perceived the creative group intervention of MBDMT as too unusual, and involvement in the therapeutic group as emotionally overwhelming. In a clinical context, an individual DMT approach could have been more suitable for these patients.

The evaluation of *acceptability of outcome measures* indicated that in comparison with HADS, PHQ-9 could show a higher sensitivity in assessing symptoms of depression for this patient group. The construction of the HADS relies on anhedonia, not on somatic symptoms, and it is sensitive to mild distress as it excludes symptoms of severe mental illness. PHQ-9 is constructed as a diagnostic tool for clinical depression and strongly correlates with mental and physical health difficulties (e.g., self-reported disability days and clinical visits) (Anderson et al., 2011), making it an appropriate tool for use within this study. Furthermore, PHQ-9 was evaluated by patients with unexplained medical conditions as a more appropriate measurement tool than CORE or HADS in a TBMA pilot study (Payne, personal communication, December 2020). Finally, chronic pain patients are often more interested in the physical representation of their difficulties than the emotional aspects. It, therefore, appeared that PHQ-9 was a more suitable measure of depression for this study, as it captured changes in the somatic aspects of psycho-emotional distress.

Similary, the General Anxiety Disorder (GAD-7) scale was considered a good tool to assess the anxiety level for the chronic pain patient group. GAD-7 has good psychometric properties and is sensitive as a clinical outcome measure (National IAPT Programme Team, 2011). Both GAD-7 and PHQ-9 were also used as measurement tools for depression and anxiety in previous research studies with a similar population in TBMA (Payne and Brooks, 2016, 2017). It was, therefore, decided to use this tool along with PHQ-9 instead of HADS for the larger scale clinical trial following the pilot.

Somatic sensitivity may also be relevant to the mindfulness measure. In the present study, the FFMQ was used to assess changes in mindfulness aspects. The FFMQ evaluates the components of dispositional mindfulness, i.e., the tendency to express mindful attitudes and behaviors in everyday life (observing experience, using language to describe experience, acting with awareness, being non-reactive, and being non-judgmental). However, the FFMQ does not distinguish between attention directed to exteroception, interoception, or thoughts (Hanley et al., 2017). Since body awareness is one of the basic components of the MBDMT intervention, a measurement that detects the aspects of interoceptive body awareness may be more appropriate in future studies. The Multidimensional Assessment of Interoceptive Awareness (MAIA, Mehling et al., 2012) is one such example: it allows an assessment of awareness of the body's physiological condition alongside the evaluative interpretations arising in tandem with that awareness (Mehling, 2016). Mehling et al. (2012) also stated that the MAIA can be helpful in researching mind–body interventions, where the multidimensional assessment of body awareness can be used to understand which aspects of body awareness contribute to improvements in clinical outcomes. MAIA has been used as a body awareness assessment tool in a previous research on the use of DMT for chronic pain patients (Shim, 2015b).

Mindfulness scores did not show statistically significant changes after the 5-week-long MBDMT intervention. The most commonly used structure in MBI research is 8 weeks, with a group session once a week and an emphasis on regular practice at home (Bawa et al., 2015). The present length of the MBDMT intervention may not be enough to develop sustainable mindfulness skills. The “dosage” of this DMT intervention might have been insufficient to create quantitatively observable changes in mindfulness outcome measures. Another possible interpretation is that the working mechanisms of the MBDMT model were more closely connected with active therapeutic factors in DMT than with mindfulness interventions. To research this hypothesis, future studies should involve qualitative and quantitative analyses of therapeutic mechanisms. Future research may also attempt to answer questions present in the DMT literature (Koch et al., 2019): what do DMT and mindfulness-based interventions have in common? Additional mindfulness principles that have been incorporated in DMT through the practice of authentic movement, such as the concept of bodymindfulness (Payne and Brooks, 2019) or “bodyfulness” (Caldwell, 2014), can be further considered in future research studies.

At the time of writing, to the best of our knowledge, this is the first pilot clinical trial to explore a mindfulness-based model in DMT for the specific patient group. There are several publications available at the time of writing this paper: a published case analysis, in which the principles of mindfulness are used within the context of somatic psychotherapy and DMT as a pathway toward embodiment (Tantia, 2013); a research study showing mindfulness skill training as one of the guiding principles of therapeutic intervention for patients with depression (Pylvänäinen et al., 2015); and therapy models that offer the perspective of mindfulness meditations and mindful movement practices, e.g., tai chi and yoga, as an important aspect of the DMT intervention (Barton, 2011; Sanchez, 2012; Olmedo, 2020). TBMA® created by Payne (2009a,b) is an integrative approach underpinned by principles of experiential learning cycles, dance movement psychotherapy, and mindfulness research, created and researched for patients with medically unexplained symptoms (Payne et al., 2020). However, the MBDMT is the first model in DMT to be adapted for chronic pain patients that employs aspects from other models in a unique way. The model uses mindfulness as an integral part of the therapeutic process, in which the methodology of mindfulness practice and the understanding of the working mechanisms are integrated within the creative process of DMT. The MBDMT model adopts a bi-directional body–mind approach, in which awareness of the body is used as a physical portal to consciousness (Eddy, 2016), and awareness of the mind is seen as a metacognitive state—ability to observe, explore, and gain the understanding of the processes and relationships between mind and body, with both aspects (i.e., body awareness and meta-cognitive aspects) of mindfulness being equally important (Majore-Dusele and Karkou, 2018). In comparison with other mindfulness-based interventions, the advantage of the MBDMT is that mindfulness is put into action through creative activity, and creative methods enable the participant to fully experience and to be able to observe the experience at the same time.

### Limitations and Future Research Directions

The first limitation of the study is the small sample size (*n* = 29) that reduces the generalizability and statistical power of calculations and questions the reliability of the quantitative findings. Qualitative data were used to complement the findings coming from quantitative data and to strengthen the conclusions regarding the study's feasibility and the impact of the intervention on specific self-reported measures completed by the participants. The use of self-reported scales is another limitation of this study. One of the clinical characteristics of chronic pain patients includes alexithymia—a decreased ability to identify and describe emotional states and differentiate them from bodily sensations. This questions the participants' ability to evaluate their own psycho-emotional states properly. Although the validity and reliability of the self-report scales used were high, in future research, both subjective and objective measures should be incorporated to increase the validity of the research findings. Another limitation is the fact that there were only female participants in the study, questioning the external validity of the study and suggesting a potential selection bias. In addition, the length of the intervention (10 sessions) and the fact that the therapeutic process was directed by a dance movement therapist in training should be considered as possible limitations of the study and as factors with capacity to influence the results of the study.

In contrast, the strength of the study is based on the use of randomization and the presence of homogeneity of the study's sample. Further strengths can be assigned to the careful development of the intervention integrating the two practices.

## Conclusions

The MBDMT is a feasible and promising intervention for chronic pain patients. The participants in the MBDMT group reported a significant decrease in pain intensity in comparison with the TAU control group in both post-intervention measures. A decrease in depression symptoms was significant in post-intervention measurement. Anxiety also changed in the expected direction for the MBDMT group. Although the results did not reach statistical significance, there was reliable improvement for 73% of the patients attending the intervention group. Scores on mindfulness measures changed in the expected direction for the MBDMT group, but did not reach statistical significance when compared with scores from the control group on the same measures.

Still, it was concluded that the pilot study offered sufficient information and preliminary results to enable the researchers to move to an RCT stage. It is expected that a larger sample, the inclusion of an active control, the replication of the group to more than one site delivered by more than one therapist, and the inclusion of additional somatic outcome measures will strengthen this pilot study enabling generalizable findings on the effectiveness of this approach to people with chronic pain.

## Data Availability Statement

The datasets generated for this study are available on request to the corresponding author.

## Ethics Statement

The studies involving human participants were reviewed and approved by The Ethics Committee of Riga Stradins University. The patients/participants provided their written informed consent to participate in this study.

## Author Contributions

IM-D was responsible for the conception and design of the study, data collection, interventions implementation, data analysis and interpretation, and writing the manuscript. VK was responsible for the conception and design of the study and contributed to the data analysis, interpretation, and manuscript development. IM contributed to the interpretation of the data and manuscript development. All authors have approved the final version of the manuscript.

## Conflict of Interest

The authors declare that the research was conducted in the absence of any commercial or financial relationships that could be construed as a potential conflict of interest.

## Footnotes

^1^Thematic Network on the Societal Impact of Pain, 2018: https://ec.europa.eu/health/sites/health/files/policies/docs/ev_20181112_co07_en.pdf.

^2^IASP, 2019: https://www.iasp-pain.org/PublicationsNews/NewsDetail.aspx?ItemNumber=8340&navItemNumber=643.

^3^ICD-11, 2018: https://icd.who.int/browse11/l-m/en.

^4^European Association of Dance Movement Therapy, 2013: https://www.eadmt.com/?action=article&id=22.

## References

[B1] Ak I. Sayar K. Yontem T. (2004). Alexithymia, somatosensory amplification and counter-dependency in patients with chronic pain. Pain Clin. 16, 43–51. 10.1163/15685690432285869310849453

[B2] Allais G. Chiarle G. Sinigaglia S. Airola G. Schiapparelli P. Benedetto C. (2020). Gender-related differences in migraine. Neurol. Sci. 41, 429–436. 10.1007/s10072-020-04643-8 32845494 PMC7704513

[B3] Anderson J. K. Zimmerman L. Caplan L. Michaud K. (2011). Measures of rheumatoid arthritis disease activity: patient (PtGA) and Provider (PrGA) Global Assessment of Disease Activity, Disease Activity Score (DAS) and Disease Activity Score with 28-Joint Counts (DAS28), Simplified Disease Activity Index (SDAI), Cl. Arthritis Care Res. 63, 14–36. 10.1002/acr.20621 22588741

[B4] Ashina S. Lipton R. B. Bendtsen L. Hajiyeva N. Buse D. C. Lyngberg A. C.. (2018). Increased pain sensitivity in migraine and tension-type headache coexistent with low back pain: a cross-sectional population study. Eur. J. Pain 22, 904–914. 10.1002/ejp.1176 29349847

[B5] Bäckryd E. Persson E. B. Larsson A. I. Fischer M. R. Gerdle B. (2018). Chronic pain patients can be classified into four groups: clustering-based discriminant analysis of psychometric data from 4665 patients referred to a multidisciplinary pain centre (a SQRP study). PLoS ONE 13:e0192623. 10.1371/journal.pone.0192623 29420607 PMC5805304

[B6] Baer R. A. Smith G. T. Hopkins J. Krietemeyer J. Toney L. (2006). Using self-report assessment methods to explore facets of mindfulness. Assessment 13, 27–45. 10.1177/1073191105283504 16443717

[B7] Barton E. J. (2011). Movement and mindfulness: a formative evaluation of a dance/movement and yoga therapy program with participants experiencing severe mental illness. Am. J. Dance Therap. 33, 157–181. 10.1007/s10465-011-9121-7

[B8] Bawa F. L. Mercer S. W. Atherton R. J. Clague F. Keen A. Scott N. W.. (2015). Does mindfulness improve outcomes in patients with chronic pain? Systematic review and meta-analysis. Br. J. General Practice 65, 387–400. 10.3399/bjgp15X685297 26009534 PMC4439829

[B9] Bjelland I. Dahl A. A. Haug T. T. Neckelmann D. (2002). The validity of the hospital anxiety and depression scale: an updated literature review. J. Psychosom. Res. 52, 69–77. 10.1016/S0022-3999(01)00296-3 11832252

[B10] Bojner-Horwitz E. Theorell T. Anderberg U. M. (2003). Dance/movement therapy and changes in stress-related hormones: a study of fibromyalgia patients with video-interpretation. Arts Psychotherap. 30, 255–264. 10.1016/j.aip.2003.07.001

[B11] Borsook D. Youssef A. M. Simons L. Elman I. Eccleston C. (2018). When pain gets stuck: the evolution of pain chronification and treatment resistance. Pain 159, 2421–2436. 10.1097/j.pain.0000000000001401 30234696 PMC6240430

[B12] Burns J. W. (2016). Mechanisms, mechanisms, mechanisms: it really does all boil down to mechanisms. Pain 157:2393. 10.1097/j.pain.0000000000000696 27548048 PMC5846486

[B13] Caldwell C. (2014). Mindfulness & bodyfulness: a new paradigm. J. Contemplative Inquiry. 1:1.

[B14] Cramer H. Haller H. Lauche R. Dobos G. (2012). Mindfulness-based stress reduction for low back pain. a systematic review. BMC Complement. Alternat. Med. 12, 1–8. 10.1186/1472-6882-12-162 23009599 PMC3520871

[B15] Davis D. A. Luecken L. J. Zautra A. J. (2005). Are reports of childhood abuse related to the experience of chronic pain in adulthood? A meta-analytic review of the literature. Clin. J. Pain 21, 398–405. 10.1097/01.ajp.0000149795.08746.31 16093745

[B16] Dworkin R. H. Turk D. C. Farrar J. T. Haythornthwaite J. A. Jensen M. P. Katz N. P.. (2005). Core outcome measures for chronic pain clinical trials: IMMPACT recommendations. Pain 113, 9–19. 10.1016/j.pain.2004.09.012 15621359

[B17] Eddy M. (2016). Mindful Movement: The Evolution of the Somatic Arts and Conscious Action. Chicago, IL: Intellect, The University of Chicago Press.

[B18] Erber B. (2015). Growing from pain–a creative process. Pain Rehabil. J. Physiotherap. Pain Assoc. 2015, 11–16.

[B19] Farrar J. T. Young J. P. LaMoreaux L. Werth J. L. Poole R. M. (2001). Clinical importance of changes in chronic pain intensity measured on an 11-point numerical pain rating scale. Pain 94, 149–158. 10.1016/S0304-3959(01)00349-9 11690728

[B20] Fayaz A. Croft P. Langford R. M. Donaldson L. J. Jones G. T. (2016). Prevalence of chronic pain in the UK: a systematic review and meta-analysis of population studies. BMJ Open 6:6. 10.1136/bmjopen-2015-010364 27324708 PMC4932255

[B21] Gatchel R. J. Bo Peng Y. Peters M. L. Fuchs P. N. Turk D. C. (2007). Biopsychosocial approach to chronic pain. Psychol. Bull. 133, 581–624. 10.1037/0033-2909.133.4.581 17592957

[B22] Goldberg D. S. McGee S. J. (2011). Pain as a global public health priority. BMC Public Health 11, 1–5. 10.1186/1471-2458-11-770 21978149 PMC3201926

[B23] Grossman P. Kappos L. Gensicke H. D'Souza M. Mohr D. C. Penner I. K.. (2010). MS quality of life, depression, and fatigue improve after mindfulness training: a randomized trial. Neurology 75, 1141–1149. 10.1212/WNL.0b013e3181f4d80d 20876468 PMC3463050

[B24] Guhn M. Forer B. Zumbo B. D. (2014). Reliable change index, in Encyclopedia of Quality of Life and Well-Being Research, ed Michalos A. C. (Dordrecht: Springer), 5459–5462. 10.1007/978-94-007-0753-5_2465

[B25] Hägg O. Fritzell P. Nordwall A. (2003). The clinical importance of changes in outcome scores after treatment for chronic low back pain. Eur. Spine J. 12, 12–20. 10.1007/s00586-002-0464-0 12592542

[B26] Hanley A. W. Mehling W. E. Garland E. L. (2017). Holding the body in mind: Interoceptive awareness, dispositional mindfulness and psychological well-being. J. Psychosom. Res. 99, 13–20. 10.1016/j.jpsychores.2017.05.014 28712417 PMC5522814

[B27] Hansson M. Chotai J. Nordstöm A. Bodlund O. (2009). Comparison of two self-rating scales to detect depression: HADS and PHQ-9. Br. J. General Practice 59, 283–288. 10.3399/bjgp09X454070 19761655 PMC2734374

[B28] Hülsebusch J. Hasenbring M. I. Rusu A. C. (2016). Understanding pain and depression in back pain: the role of catastrophizing, help-/hopelessness, and thought suppression as potential mediators. Int. J. Behav. Med. 23, 251–259. 10.1007/s12529-015-9522-y 26590138

[B29] Hyvönen K. Pylvänäinen P. Muotka J. S. Lappalainen R. (2020). The effects of dance movement therapy in the treatment of depression: a multi-centre, randomised controlled trial in Finland. Front. Psychol. 11:1687. 10.3389/fpsyg.2020.01687 32903394 PMC7434972

[B30] Jacobson N. S. Truax P. (1991). Clinical significance: a statistical approach to defining meaningful change in psychotherapy research. J. Consult. Clin. Psychol. 59, 12–19. 10.1037/0022-006X.59.1.12 2002127

[B31] Jiménez-Sánchez S. Fernández-de-las-Peñas C. Carrasco-Garrido P. Hernández-Barrera V. Alonso-Blanco C. Palacios-Ceña D.. (2012). Prevalence of chronic head, neck and low back pain and associated factors in women residing in the Autonomous Region of Madrid (Spain). Gaceta Sanitaria 26, 534–540. 10.1016/j.gaceta.2011.10.012 22342049

[B32] Kabat-Zinn J. (2003). Mindfulness-based interventions in context: past, present, and future. Clin. Psychol. 10, 144–156. 10.1093/clipsy.bpg016

[B33] Kabat-Zinn J. Lipworth L. Burncy R. Sellers W. (1986). Four-year follow-up of a meditation-based program for the self-regulation of chronic pain: treatment outcomes and compliance. Clin. J. Pain 2, 159–774. 10.1097/00002508-198602030-00004

[B34] Karkou V. Aithal S. Zubala A. Meekums B. (2019). Effectiveness of dance movement therapy in the treatment of adults with depression: a systematic review with meta-analyses. Front. Psychol. 10:936. 10.3389/fpsyg.2019.00936 31130889 PMC6509172

[B35] Kazdin A. E. (2009). Understanding how and why psychotherapy leads to change. Psychotherap. Res. 19, 418–428. 10.1080/10503300802448899 19034715

[B36] Keefe F. J. Rumble M. E. Scipio C. D. Giordano L. A. Perri L. M. (2004). Psychological aspects of persistent pain: current state of the science. J. Pain 5, 195–211. 10.1016/j.jpain.2004.02.576 15162342

[B37] Koch S. Kunz T. Lykou S. Cruz R. (2014). Effects of dance movement therapy and dance on health-related psychological outcomes: a meta-analysis. Arts Psychotherap. 41, 46–64. 10.1016/j.aip.2013.10.004 31481910 PMC6710484

[B38] Koch S. C. Riege R. F. F. Tisborn K. Biondo J. Martin L. Beelmann A. (2019). Effects of dance movement therapy and dance on health-related psychological outcomes. a meta-analysis update. Front. Psychol. 10:1806. 10.3389/fpsyg.2019.01806 31481910 PMC6710484

[B39] Kress H. G. Aldington D. Alon E. Coaccioli S. Collett B. Coluzzi F.. (2015). A holistic approach to chronic pain management that involves all stakeholders: change is needed. Curr. Med. Res. Opin. 31, 1743–1754. 10.1185/03007995.2015.1072088 26172982

[B40] Kroenke K. Spitzer R. L. (2002). The PHQ-9: a new depression diagnostic and severity measure. Psychiatric Ann. 32, 509–515. 10.3928/0048-5713-20020901-06

[B41] Lachapelle D. L. Hadjistavropoulos T. (2005). Age-related differences among adults coping with pain: evaluation of a developmental life-context model. Canad. J. Behav. Sci./Revue Canadienne Des Sci. Du Comportement. 37, 123–137. 10.1037/h0087250

[B42] Lambert M. J. Barley D. E. (2001). Research summary on the therapeutic relationship and psychotherapy outcome. Psychotherapy 38, 357–361. 10.1037/0033-3204.38.4.357

[B43] Lindsay E. K. Creswell J. D. (2017). Mechanisms of mindfulness training: Monitor and Acceptance Theory (MAT). Clin. Psychol. Rev. 51, 48–59. 10.1016/j.cpr.2016.10.011 27835764 PMC5195874

[B44] Majore-Dusele I. Karkou V. (2018). A mindfulness approach to dance movement therapy - in between cognitive and embodied therapies: first results from grounded theory research, in Crossing Borders and the In-Between. International Scientific Conference, 13. Retrieved from: https://www.eadmt.com/doc/eadmt-pdf.pdf (accessed July 12, 2020).

[B45] Majore-Dušele I. Millere I. Karkou V. Logina I. (2019). Development of a working model in mindfulness-based dance movement therapy. knowledge for use in practice, in Proceedings of the International conference on Medical and Health Care Science, 267. Retrieved from: http://conference2019.rsu.lv/sites/default/files/documents/knowledge_for_use_in_practice_abstracts_rev.pdf (accessed July 10, 2020).

[B46] Majore-Dušele I. Paiča I. Mārtinsone K. Millere I. (2018). Characteristics of mindfulness based interventions for different patient groups – literature review. Soc. Integrat. Educ. Proc. Int. Sci. Conference 7, 140–152. 10.17770/sie2018vol1.3298

[B47] Majors M. (2013). Adaptation of Five-facets of Mindfulness Questionnaire (in Latvian). Master's thesis, Riga Teacher Training and Educational Management Academy, Riga, LV.

[B48] Manea L. Gilbody S. McMillan D. (2012). Optimal cut-off score for diagnosing depression with the Patient Health Questionnaire (PHQ-9): a meta-analysis. CMAJ 184, 191–196. 10.1503/cmaj.110829 22184363 PMC3281183

[B49] Meekums B. Karkou V. Nelson E. A. (2015). Dance movement therapy for depression. Cochrane Database of Syst Rev. 2:CD009895. 10.1002/14651858.CD009895.pub2 PMC8928931 25695871

[B50] Mehling W. (2016). Differentiating attention styles and regulatory aspects of self-reported interoceptive sensibility. Philos. Transact. R. Soc. B Biol. Sci. 371:1708. 10.1098/rstb.2016.0013 28080970 PMC5062101

[B51] Mehling W. E. Price C. Daubenmier J. J. Acree M. Bartmess E. Stewart A. (2012). The Multidimensional Assessment of Interoceptive Awareness (MAIA). PLoS ONE 7:e0208034. 10.1371/journal.pone.0048230 PMC3486814 23133619

[B52] Melzack R. (1999). From the gate to the neuromatrix. Pain 82, 121–126. 10.1016/S0304-3959(99)00145-1 10491980

[B53] National IAPT Programme Team. (2011). The IAPT Data Handbook: Guidance on Recording and Monitoring Outcomes to Support Local Evidence-based Practice. Version 2.0.1. London: National IAPT Programme Team. Available online at: https://webarchive.nationalarchives.gov.uk/20160302160058/http://www.iapt.nhs.uk/silo/files/iapt-data-handbook-v2.pdf (accessed November 11, 2020).

[B54] Nicholas M. K. Linton S. J. Watson P. J. Main C. J. (2011). Early identification and management of psychological risk factors (“Yellow Flags”) in patients with low back pain: a reappraisal. Phys. Ther. 91, 737–753. 10.2522/ptj.20100224 21451099

[B55] Olmedo M. (2020). Moving Through Depression: Development of a Dance/Movement Therapy Method in Psychiatric Inpatient Care. master's thesis, Lesley University, Cambridge, MA.

[B56] Payne H. (2009a). Medically unexplained conditions and the bodymind approach. Counsel Primary Care Rev. 10, 6–8.

[B57] Payne H. (2009b). The BodyMind Approach (BMA) to psychotherapeutic groupwork with patients with medically unexplained symptoms (MUS): a review of the literature, description of approach and methodology for a pilot study. Eur. J. Psychotherap. Counsel. 11, 287–310. 10.1080/13642530903230392

[B58] Payne H. Brooks S. D. M. (2016). Clinical outcomes from The BodyMind Approach^TM^ in the treatment of patients with medically unexplained symptoms in primary health care in England: practice-based evidence. Arts Psychotherap. 47, 55–65. 10.1016/j.aip.2015.12.001

[B59] Payne H. Brooks S. D. M. (2017). Moving on: the BodyMind approachtm for medically unexplained symptoms. J. Public Ment. Health 16, 1–9. 10.1108/JPMH-10-2016-0052

[B60] Payne H. Brooks S. D. M. (2018). Different strokes for different folks: the bodymind approach as a learning tool for patients with medically unexplained symptoms to self-manage. Front. Psychol. 9:2222. 10.3389/fpsyg.2018.02222 30483203 PMC6243086

[B61] Payne H. Brooks S. D. M. (2019). Medically unexplained symptoms and attachment theory: the BodyMind Approach®. Front. Psychol. 10:1818. 10.3389/fpsyg.2019.01818 31780974 PMC6851196

[B62] Payne H. Brooks S. D. M. (2020). A qualitative study of the views of patients with medically unexplained symptoms on The BodyMind Approach®: employing embodied methods and arts practices for self-management. Front. Psychol. 11:3223. 10.3389/fpsyg.2020.554566 33364994 PMC7750328

[B63] Payne H. Lin Y. Cipolletta S. Winter D. (2017). Beyond mind and body: Person in (inter) action, in Personal Construct Psychology at 60: Past, Present and Future, ed Winter D. Cummins P. Procter H. (Hatfield: Cambridge Scholars Publishing), 209.

[B64] Payne H. Roberts A. Jarvis J. (2020). The bodymind approach® as transformative learning to promote self-management for patients with medically unexplained symptoms. J. Transform. Educ. 18, 114–137. 10.1177/1541344619883892

[B65] Pylvänäinen P. M. Muotka J. S. Lappalainen R. (2015). A dance movement therapy group for depressed adult patients in a psychiatric outpatient clinic: effects of the treatment. Front. Psychol. 6:980. 10.3389/fpsyg.2015.00980 26217292 PMC4498018

[B66] Reiner K. Tibi L. Lipsitz J. D. (2013). Do mindfulness-based interventions reduce pain intensity? a critical review of the literature. Pain Med. 14, 230–242. 10.1111/pme.12006 23240921

[B67] Rustøen T. Wahl A. K. Hanestad B. R. Lerdal A. Paul S. Miaskowski C. (2005). Age and the experience of chronic pain: differences in health and quality of life among younger, middle-aged, and older adults. Clin. J. Pain. 21, 513–523. 10.1097/01.ajp.0000146217.31780.ef 16215337

[B68] Saariaho A. S. Saariaho T. H. Mattila A. K. Karukivi M. R. Joukamaa M. I. (2013). Alexithymia and depression in a chronic pain patient sample. Gen. Hosp. Psychiatry. 35, 239–245. 10.1016/j.genhosppsych.2012.11.011 23333032

[B69] Sanchez M. A. (2012). Mindful Bodies: The Use of Guided Meditation with Dance/Movement Therapy in Addiction Treatment. master's thesis, Columbia College Chicago, Chicago, IL.

[B70] Shapiro S. L. Carlson L. E. Astin J. A. Freedman B. (2006). Mechanisms of mindfulness. J. Clin. Psychol. 62, 373–386. 10.1002/jclp.20237 16385481

[B71] Shim M. (2015a). (541) Factors and mechanisms of dance/movement therapy for resilience-building in people living with chronic pain. J. Pain 16:111. 10.1016/j.jpain.2015.01.462

[B72] Shim M. (2015b). A Model of Dance/Movement Therapy for Resilience-Building in People Living With Chronic Pain: A Mixed Methods Grounded Theory Study. dissertation thesis, Drexel University, Philadelphia, PA.

[B73] Shim M. Johnson R. B. Gasson S. Goodill S. Jermyn R. Bradt J. (2017). A model of dance/movement therapy for resilience-building in people living with chronic pain. Eur. J. Integr. Med. 9, 27–40. 10.1016/j.eujim.2017.01.011

[B74] Šmite D. Ancāne G. (2010). Psychosomatic aspects of chronic low back pain syndrome. Proce. Latvian Acad. Sci. B 64, 202–208. 10.2478/v10046-011-0005-510231809

[B75] Tantia J. F. (2013). Mindfulness and dance/movement therapy for treating trauma, in Mindfulness in the Creative Arts Therapies: Theory and Practice, ed. Rappaport L. (London: Jessica Kingsley), 96–107.

[B76] Treede R. D. Rief W. Barke A. Aziz Q. Bennett M. I. Benoliel R.. (2019). Chronic pain as a symptom or a disease: the IASP classification of chronic pain for the International Classification of Diseases (ICD-11). Pain 160, 19–27. 10.1097/j.pain.0000000000001384 30586067

[B77] Turner J. A. Anderson M. L. Balderson B. H. Cook A. J. Sherman K. J. Cherkin D. C. (2016). Mindfulness-based stress reduction and cognitive-behavioral therapy for chronic low back pain: similar effects on mindfulness, catastrophizing, self-efficacy, and acceptance in a randomized controlled trial. Pain 157: 2434. 10.1097/j.pain.0000000000000635 27257859 PMC5069124

[B78] Veehof M. M. Trompetter H. R. Bohlmeijer E. T. Schreurs K. M. G. (2016). Acceptance- and mindfulness-based interventions for the treatment of chronic pain: a meta-analytic review. Cogn. Behav. Ther. 45, 5–31. 10.1080/16506073.2015.1098724 26818413

[B79] Williams A. Eccleston C. Morley S. (2012). Cochrane review psychological therapie for the management of chronic pain (excluding headache) in adults. Cochrane Database Syst. Rev. 11:CD007407. 10.1002/14651858.CD007407.pub3 23152245 PMC6483325

[B80] Xu Y. Wang Y. Chen J. He Y. Zeng Q. Huang Y.. (2020). The comorbidity of mental and physical disorders with self-reported chronic back or neck pain: Results from the China Mental Health Survey. J. Affect. Disord. 260, 334–341. 10.1016/j.jad.2019.08.089 31521871

[B81] Zigmond A. S. Snaith R. P. (1983). The hospital anxiety and depression scale. Acta Psychiatr. Scand. 67, 361–370. 10.1111/j.1600-0447.1983.tb09716.x 6880820

